# 

**Published:** 1872-05

**Authors:** 


☞ Should any of our Subscribers or Exchanges fail to get THE COMPANION regularly, they will confer a favor by notifying us of the fact.

☞ Our Mailing Clerk will faithfully forward THE COMPANION. Subscribers will please notify Post-masters that it will reach their offices, if not lost on the way, between the 15th and 20th of every month. Should it fail to come, after a reasonable time, notify the Editors, and it will be sent.

☞ We respectfully solicit Original Articles, Reports of Societies, Clinics, Home and Foreign Correspondents, News, etc., etc., of interest to medical readers.

☞ To insure publication, articles should be practical, brief as possible, and carefully written, on ONE side of the paper. Articles should be received by the 15th of one month to insure publication in the next. ☞ Friends, send in your articles. ☜

☞ All Subscribers and others will please write their names, post-offices, county and State *plainly.* ☞ *Remember, this is now required by the Post-Master General.*

---

TO OUR SUBSCRIBERS.—Should any mistakes occur, either in name, post-office, or otherwise, subscribers are requested to address Mr. J. J. TOON, P. O. Box, No. 24. All errors will be corrected upon notification.

THANKS.—Our thanks are due our friends for their contributions to our journal. The present number will bear witness to the zeal of our friends in furnishing articles for the good of the profession, and the advancement of science. We earnestly trust that all our friends will aid in so glorious a work. We have a number of communications on hand which will appear in the regular order of arrival. Do not forget to send your articles, friends. We will be pleased and happy to give you room in THE COMPANION.

PAY UP!—Many of our subscribers of last year, as well as those of the present, have not paid for their copies. Do not delay the matter, friends. If the reader should happen to be one of these, will he not send the amount due at once? Don't forget this. The price of subscription is very low. Certainly every one can send so small an amount. Do try!

### BOOKS AND PAMPHLETS.

PLAIN TALK ABOUT INSANITY, ITS CAUSES, FORMS, SYMPTOMS, AND THE TREATMENT OF MENTAL DISEASES, WITH REMARKS ON HOSPITALS AND ASYLUMS, AND THE MEDICO—LEGAL ASPECT OF INSANITY. By T. W. FISHER, M.D., late of Boston Hospital for the Insane. Boston; Alexander Moore, 1872.

This is a very readable volume, and furnishes the professional reader with many facts and suggestions of the greatest value. It is written in an easy, practical style, giving a freshness to the subject not often seen in works of like character. It will form a valuable addition to every medical library.

DISEASES OF THE SKIN: THE RECENT ADVANCES IN THEIR TREATMENT. By B JOY JEFFRIES, A. M., M. D. Boston: Alexander Moore, 1871.

This is a valuable book of 79 pages, the matter of which was originally published in *The American Journal of Syphilography and Dermatology.* It will be found a useful and instructive volume, containing much of a practical character, in a form convenient for easy reference.

ANIMAL AND VEGETABLE PARASITES OF THE HUMAN SKIN AND HAIR. By B. JOY JEFFRIES, A. M., M. D. Boston: Alexander Moore, 1872.

A very useful and instructive volume. It will be read with pleasure and profit by the physician no less than the civilian, everywhere. We commend it to the profession.

THE PHYSIOLOGICAL AND THERAPEUTICAL ACTION OF THE BROMIDE OF POTASSIUM AND BROMIDE OF AMMONIUM. In two parts. By EDWARD H. CLARKE, M. D., and ROBERT AMORY, M. D. Boston: James Campbell, 1872.

We are indebted to the publisher for the above. The work is designed to give at one view the most important points in the physiological and therapeutical action of these Bromides. Many facts of the greatest value are brought to view, making an extremely interesting and practical volume—one our readers would do well to secure.

SMALL-POX, THE PREDISPOSING CONDITIONS AND THEIR PREVENTION. By DR. CARL BOTH. Boston: Alexander Moore, 1872.

Dr. Both's monograph will be found interesting, particularly in view of the prevalence of small-pox in our country at this time. Its prevention should be a matter of earnest thought, and the fearful malignancy of the present epidemic in various parts of the Union should induce the profession to give the subject a thorough review and study.

MEMORANDA ON POISONS. By the late THOMAS HAWKS TANNER, M. D., F. L. S. Third and completely revised edition. Philadelphia: Lindsay & Blakiston, 1872.

Everything from the pen of Tanner bears the marks of thought and talent, and the above little volume is no exception to the remark. These memoranda will be found of great service to the busy practitioner and will place in his way a fund of practical information which he may be called upon to make available at any moment. Price 75 cents.

DR. RIGBY'S OBSTETRIC MEMORANDA. Fourth Edition, revised and enlarged. By ALFRED MEADOWS, M. D., Physician to the General Lying-In Hospital for Women, etc., etc. Philadelphia: Lindsay & Blakiston, 1872.

This is a valuable little book, which has reached its fourth edition, the three former, though large, having been exhausted. Dr. Meadows has made changes for the better, and added new matter to enhance the value of the volume. It will be found an excellent pocket companion for the busy practitioner, furnishing many valuable hints and affording information in times of emergency. The formulæ are alone worth the price—50 cents.

CLINICAL OBSERVATIONS ON THE DEMENTIA AND THE HEMIPLEGIA OF SYPHILIS. By M. H. HENRY, M. D., Surgeon to the New York Dispensary, etc., etc. New York: F. W. Christern, No. 72 University Place, 1872.

There is no better authority upon the subjects embraced in the above monograph, than its author. Those of our readers who feel an interest in the "dementia and hemiplegia of syphilis" will do well to secure a copy.

TREATMENT OF FEVER AND INFLAMMATION. An Essay on the typography and prevailing diseases of New Hanover, N. C. Read before the annual meeting of the North Carolina Medical Society, 1870. By S. S. Satchwell, A.M., M.D.

AN ADDRESS ON THE WELFARE OF THE MEDICAL PROFESSION. Delivered before the State Medical Society, 1868. By the President, S. S. Satchwell, M. D.

ADDRESS ON IMMIGRATION. Delivered before the North Carolina Immigration Association, by the President, S. S. Satchwell.

We return our thanks to the author for the above pamphlets. Dr. Satchwell, one of our associates of North Carolina, is a writer of great force, and a reasoner of no ordinary power. His pamphlets show this. Among the man able medical men of his State, he ranks with the very foremost.

The first-named pamphlet embodies a reply to an address of Dr. Norcom, read before the State Society and published a few months since in the COMPANION. It is manly and dignified. When two such men as Norcom and Satchwell discuss medical topics they cannot fail to shed light upon medicine As we have published Dr. Norcom's address, our friends of North Carolina solicit the publication also of Dr. Satchwell's rejoinder. As the discussion is one purely for the professional weal, and by two gentlemen regardful of that courtesy which seeks truth alone as its object, we will yield to the solicitations of our friends, and publish Dr. Satchwell's views at an early day—opening our journal to Dr. Norcom should he desire to be heard again. The ablest minds of the profession of both continents are arrayed against each other upon the subjects embraced in the addresses of our North Carolina friends.

